# The effect of deep learning reconstruction on abdominal CT densitometry and image quality: a systematic review and meta-analysis

**DOI:** 10.1007/s00330-021-08438-z

**Published:** 2021-12-15

**Authors:** J. Abel van Stiphout, Jan Driessen, Lennart R. Koetzier, Lara B. Ruules, Martin J. Willemink, Jan W. T. Heemskerk, Aart J. van der Molen

**Affiliations:** 1 Department of Clinical Technology, Faculty of Mechanical, Maritime, and Materials Engineering (3ME), Delft University of Technology, Mekelweg 2, NL-2628 CD Delft, The Netherlands; 2 Department of Radiology, Stanford University School of Medicine, 300 Pasteur Drive, S-072, Stanford, CA 94305-5105 USA; 3 Department of Radiology C-2S, Leiden University Medical Center, Albinusdreef 2, NL-2333 ZA Leiden, The Netherlands

**Keywords:** Tomography, x-ray computed, Abdomen, Image processing, computer-assisted, Deep learning

## Abstract

**Objective:**

To determine the difference in CT values and image quality of abdominal CT images reconstructed by filtered back-projection (FBP), hybrid iterative reconstruction (IR), and deep learning reconstruction (DLR).

**Methods:**

PubMed and Embase were systematically searched for articles regarding CT densitometry in the abdomen and the image reconstruction techniques FBP, hybrid IR, and DLR. Mean differences in CT values between reconstruction techniques were analyzed. A comparison between signal-to-noise ratio (SNR) and contrast-to-noise ratio (CNR) of FBP, hybrid IR, and DLR was made. A comparison of diagnostic confidence between hybrid IR and DLR was made.

**Results:**

Sixteen articles were included, six being suitable for meta-analysis. In the liver, the mean difference between hybrid IR and DLR was − 0.633 HU (*p* = 0.483, SD ± 0.902 HU). In the spleen, the mean difference between hybrid IR and DLR was − 0.099 HU (*p* = 0.925, SD ± 1.061 HU). In the pancreas, the mean difference between hybrid IR and DLR was − 1.372 HU (*p* = 0.353, SD ± 1.476 HU). In 14 articles, CNR was described. In all cases, DLR showed a significantly higher CNR. In 9 articles, SNR was described. In all cases but one, DLR showed a significantly higher SNR. In all cases, DLR showed a significantly higher diagnostic confidence.

**Conclusions:**

There were no significant differences in CT values reconstructed by FBP, hybrid IR, and DLR in abdominal organs. This shows that these reconstruction techniques are consistent in reconstructing CT values. DLR images showed a significantly higher SNR and CNR, compared to FBP and hybrid IR.

**Key Points:**

- *CT values of abdominal CT images are similar between deep learning reconstruction (DLR), filtered back-projection (FBP), and hybrid iterative reconstruction (IR).*
- *DLR results in improved image quality in terms of SNR and CNR compared to FBP and hybrid IR images.*
- *DLR can thus be safely implemented in the clinical setting resulting in improved image quality without affecting CT values.*

**Supplementary Information:**

The online version contains supplementary material available at 10.1007/s00330-021-08438-z.

## Introduction

Computed tomography (CT) acquires images of tissues inside the human body. Photons, emitted by an X-ray tube, interact with human tissue and either get absorbed due to the photoelectric effect or get scattered due to Compton scattering. A fraction of the initial photon beam leaves the patient’s body and is detected on the opposite side of the CT X-ray tube. The ratio between the incident intensity and the emerging intensity is related to the attenuation coefficient, which forms a material-specific property. Hence, by reconstructing the attenuation coefficient for each voxel (a pixel within a 3-dimensional image), imaging and identification of tissues can be carried out in a non-invasive way. However, since attenuation coefficients of various soft tissues have values close to each other, especially in the abdomen, the Hounsfield unit (HU) was introduced, which is a measure relative to the density of water. These CT values, expressed in HU, are calculated by the following formula:

$$1000* \frac{{\mu }_{\mathrm{tissue}}- {\mu }_{\mathrm{water}}}{{\mu }_{\mathrm{water}}}.$$

To reconstruct attenuation measurements by the CT scanner into HUs and display these in a comprehensible image suitable for clinical diagnosis, a method for iterative data reconstruction was proposed. This technique simulated the CT system, and based upon this model, it iteratively adjusted measured data for various factors influencing the attenuation measurements as e.g., estimated dose or noise effects. However, due to large amounts of data and the absence of sufficient computational power, iterative reconstruction (IR) was not competent for clinical practice [1].

Instead, the less computational demanding, and hence much faster, reconstruction technique filtered back-projection (FBP) was introduced. This reconstruction technique has been the most used technique for decades, until the awareness of radiation-induced health effects, such as the formation of neoplasms, grew among society. Hence, a new reconstruction technique got introduced that allowed for low-dose CT measurements while maintaining image quality in terms of signal-to-noise ratio (SNR) and contrast-to-noise ratio (CNR) [2]. This reconstruction technique resembles the initially proposed IR method, but is a combination of FBP and full IR, hence called hybrid IR. However, radiologists occasionally assess CT images reconstructed by hybrid IR as being too artificial-looking, because hybrid IR does not model the complete CT system [3–5]. Nonetheless, recent developments in computing power and the use of artificial intelligence have made it possible to apply full IR. Full IR is also referred to as model-based IR (MBIR), as it incorporates a model that simulates the CT system, including its photon beam formation principle and several factors that may deform it, due to e.g. the focal spot size or the beam hardening effect. Characteristic for MBIR is the use of backward and forward projection steps during each iteration cycle. This method enables optimizing the true data based on comparisons with estimated artificial data, which is updated for each new cycle. These comparisons are iterated until no corrections need to be made in the true data, or until the maximum number of iterations has been reached [4].

Deep learning reconstruction (DLR) uses this base of MBIR to further improve image quality. Deep learning is a subset of artificial intelligence and uses convolutional neural networks (CNNs) to learn from the input data itself. These CNNs are trained on high-quality labelled CT images and learn from unlabelled data in clinical settings just like radiologists are trained on the job. The CNNs can differentiate even better between noise and signal in comparison to FBP or hybrid IR, and therefore, even more dose reduction is possible while maintaining image quality and detail preservation [6]. DLR techniques are now being introduced into clinical practice, but whether these algorithms are applied correctly still needs to be investigated carefully. Especially for its application in abdominal CT scans—in which low contrast resolution is essential—it is important that CT values are reconstructed correctly. As DLR simulates the complete CT system, contrary to FBP and hybrid IR techniques, one might assume that CT reconstructions made by DLR may result in different CT values for certain tissues than with the use of FBP or hybrid IR. This can lead to serious complications when CT values reconstructed by DLR differ much from FBP or hybrid IR, that established cut-off-values in certain diagnostic methods will not hold anymore for DLR reconstructed CT images resulting in missing diagnoses [7]. Hence, in this systematic review and meta-analysis, we sought to evaluate differences between DLR reconstructed CT values and FBP or hybrid IR reconstructed CT values. Furthermore, we compared the image quality of these three reconstruction techniques.

## Methods

This systematic review and meta-analysis were performed according to the Preferred Reporting Items for Systematic Reviews and Meta-Analyses (PRISMA) guidelines [8].

### Literature search

PubMed and Embase databases were searched to find studies that describe the performance of one or more CT image reconstruction techniques. The following search terms were used: (“CT Densitometry” OR (“Densitometry” AND “Tomography, X-Ray Computed”)) AND “Hounsfield Units” AND (“Filtered Back Projection” OR “Iterative Reconstruction”) AND (“Artificial Intelligence” OR “Deep Learning”) AND “Abdomen”. The complete search string is described in Appendix A. No beginning search date was set; the literature search was updated until May 10, 2021.

### Exclusion criteria

The titles and abstracts of all studies were independently and blindly screened by two researchers. If the two researchers did not agree about inclusion or exclusion, a final decision was made by a third researcher. Papers were excluded if the title clearly indicated that CT densitometry was not described.

The exclusion criteria for abstract screening were as follows: (1) FBP, hybrid IR, or DLR were not described; (2) articles about segmentation; (3) not specified to the abdomen; (4) no densitometry described; and (5) full text not available in English or Dutch. The exclusion criteria for full-text eligibility were as follows: (1) no CT values, SNR, or CNR described and (2) no DLR on abdominal organs described.

### Quality assessment

All included studies were assessed with a custom-made quality assessment (QA). The aspects of the QA scale were valuable for assessing quality to all authors. A score with a range of 0–18 was assigned to each study. Points were scored for the following aspects: (1) study design; (2) data collection; (3) samples; (4) statistical analysis; (5) funding; (6) material description; (7) DLR vs FBP/IR comparison; and (8) region of interest (ROI) description. This list also contains aspects of other QA scales for quantitative studies [9]. A point was awarded if the answer to the question was “Yes”. A list of all questions is given in Appendix B. Those with a score of 6 or lower were classified as low-quality studies, 7–11 as moderate-quality studies, and 12 or more as high-quality studies.

### Descriptive analysis

Descriptive data were extracted independently by two authors. An overview of differences in CT values, SNR, and CNR between reconstruction techniques was given. Study-specific variations were reported. Data on subjective image quality in the liver was extracted by one author. The average diagnostic confidence of hybrid IR and DLR of one vendor was assessed by two radiologists on a scale from 1 to 5, where 1 = unacceptable, 2 = suboptimal, 3 = acceptable, 4 = good, and 5 = excellent.

### Data extraction

Data of CT values were only extracted for the meta-analysis if the vendor was used in more than one included study. To rule out variations between vendor’s reconstruction algorithms, one meta-analysis per vendor was made. The mean and standard deviation of the CT values were extracted for all organs that were examined in multiple included articles. When images were reviewed by more than one radiologist, the data of the most experienced one were extracted. The mean difference was calculated by extracting the CT values reconstructed by DLR from the values reconstructed by hybrid IR.

### Statistical analysis

The data were analyzed using random effects (DerSimonian-Laird method) in OpenMeta[Analyst] [10] software version 10.12. Statistical heterogeneity was evaluated by calculating *I*^2^ statistics. Low heterogeneity was defined as *I*^2^ < 50%. The statistical significance was defined as *p* < 0.05. The results were summarized in forest plots.

## Results

### Study selection

**Fig. 1:**
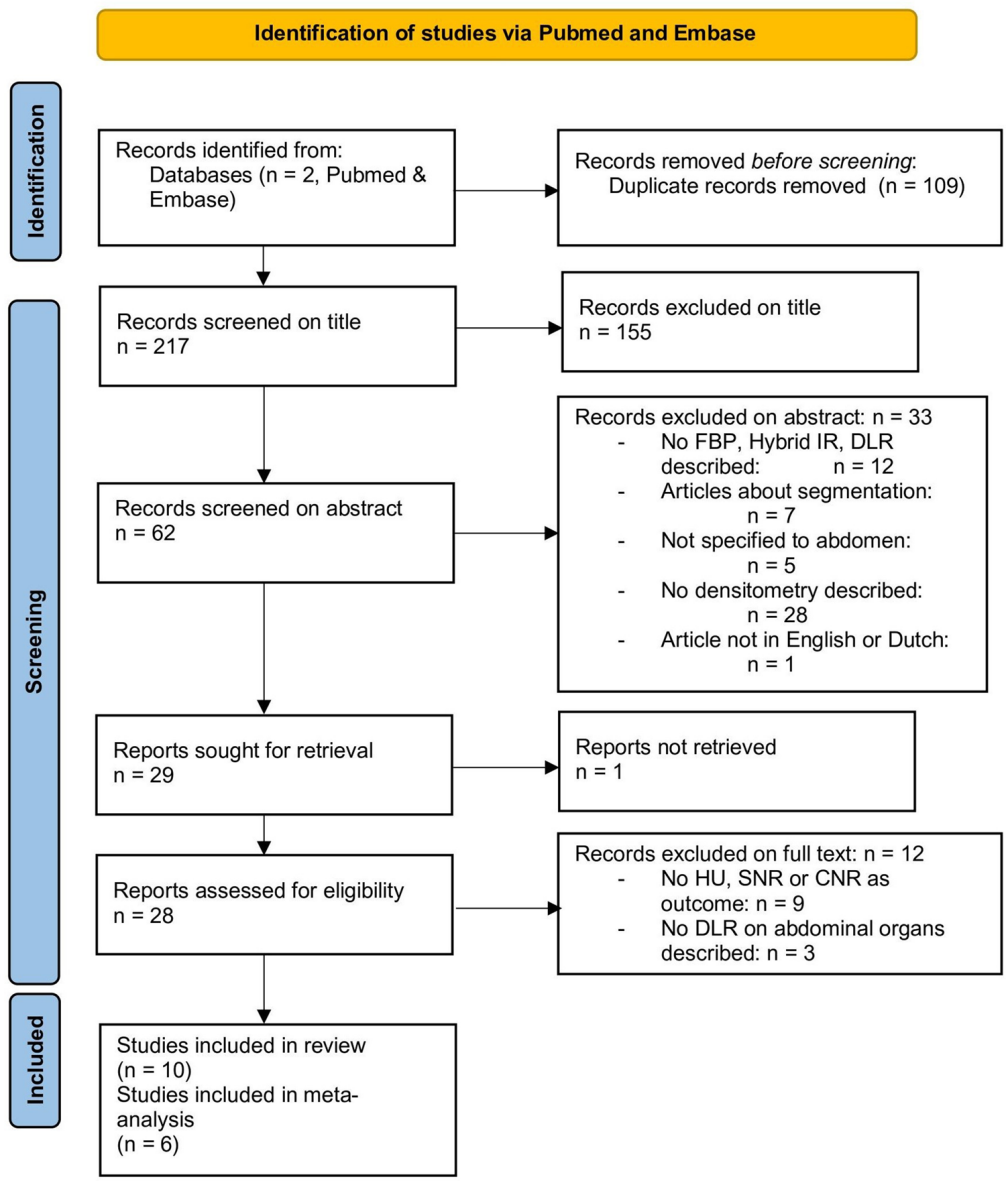
Flowchart of literature search. FBP = filtered back-projection. IR = iterative reconstruction. DLR = deep learning reconstruction. HU = Hounsfield unit. SNR = signal-to-noise ratio. CNR = contrast-to-noise ratio

The search in PubMed and Embase resulted in 178 and 148 studies, respectively. After excluding duplicated articles, a total of 217 studies were obtained. Subsequently, all titles were screened, and 155 articles were excluded. After that, abstracts were screened, and 33 studies were excluded based on the pre-determined exclusion criteria. There were several studies excluded based on more than one criterium. As a result, a total of 28 studies were full text reviewed. Among them, 9 studies were excluded due to the lack of HU, SNR, or CNR as an outcome measure, and 3 studies excluded because no DLR on abdominal organs was described. Finally, 16 articles were included in the systematic review. Of these 16 [11–26], 6 articles [13, 15–18, 21] were included in the meta-analysis (Fig. 1).

### Study characteristics

All 16 articles were either retrospective or prospective studies. All included studies compared DLR to another reconstruction technique, either a hybrid IR technique, FBP, or both. In all studies, images were reconstructed with different techniques, while using the same raw image data. Table 1 summarizes the characteristics of each study, such as the number of patients, which reconstruction techniques were compared, and what quantitative outcome measures were described.

**Table 1:** Characteristics of included studies

Author	Publication date	Study goal	Study type	Number of patients	Reconstruction methods	Vendor	Quantitative outcome measures	Abdominal organs	CNR formula
**M. Akagi **[12]	Jan 2019		RS	46	AIDR3D FIRST AiCE	Canon Medical Systems	CT value Noise CNR	Liver	$\frac{{ROI}_{\mathrm{organ}}-{ROI}_{\mathrm{muscle}}}{\mathrm{noise}}$
**M. Akagi **[11]	Oct 2020		RS	50	AIDR3D FIRST AiCE	Canon Medical Systems	Noise CNR	Liver	$\frac{{ROI}_{\mathrm{organ}}-{ROI}_{\mathrm{muscle}}}{\mathrm{noise}}$
**L. Cao **[13]	Feb 2021	Using DLR to reduce dose and improve image quality	PS	40	ASIR-V 50% DLR-H	GE Healthcare	CT value Noise CNR	Liver Spleen	$\frac{{ROI}_{\mathrm{organ}}-{ROI}_{\mathrm{muscle}}}{\mathrm{noise}}$
**Y. Ichikawa **[14]	Jan 2021		RS	50	ASIR-V DLR-H	GE Healthcare	Noise SNR/CNR	Liver	$\frac{{ROI}_{\mathrm{organ}}-{ROI}_{\mathrm{liver}}}{\mathrm{noise}}$
**C.T. Jensen **[15]	July 2020	Quantitative and qualitative evaluation of DLR	RS	40	ASIR-V 30% DLR-L/M/H	GE Healthcare	CT value Noise CNR	Liver Spleen	$\frac{{ROI}_{\mathrm{organ}}-{ROI}_{\mathrm{muscle}}}{\mathrm{noise}}$
**T. Kaga **[16]	April 2021	Evaluating image quality and lesion detection of DLR	PS	59	ASIR-V 40% DLR/L/M/H	GE Healthcare	CT value Noise SNR/CNR	Liver Spleen Pancreas	$\frac{{ROI}_{\mathrm{organ}}-{ROI}_{\mathrm{liver}}}{\mathrm{noise}}$
**J.H. Kim **[17]	Jan 2021	Evaluating image quality and DLR	RS	58	ASIR-V 30% DLR-M/H	GE Healthcare	CT value Noise SNR/CNR	Liver	$\frac{{2\left({ROI}_{\mathrm{organ}}-{ROI}_{background}\right)}^{2}}{{{SD}_{\mathrm{organ}}}^{2}+{{SD}_{\mathrm{background}}}^{2}}$
**L.L. Li **[18]	March 2021	Quantitative and qualitative evaluation of DLR	PS	47	FBP ASIR-V 40/80% DLR-M/H	GE Healthcare	CT value Noise SNR/CNR	Liver Spleen Kidney	$\frac{{ROI}_{\mathrm{organ}}-{ROI}_{\mathrm{muscle}}}{\mathrm{noise}}$
**Y. Nakamura **[19]	July 2019		RS	58	AIDR3D AiCE	Canon Medical Systems	Noise CNR	Liver	$\frac{{ROI}_{\mathrm{organ}}-{ROI}_{\mathrm{tumor}}}{\mathrm{noise}}$
**Y. Nakamura **[20]	Nov 2020		RS	72	AIDR3D FIRST AiCE	Canon Medical Systems	Noise CNR	Liver	$\frac{{ROI}_{\mathrm{organ}}-{ROI}_{\mathrm{muscle}}}{\mathrm{noise}}$
**Y. Noda **[21]	Feb 2021	Evaluating image quality and lesion detection of DLR	PS	59	ASIR-V 40% DLR-H	GE Healthcare	CT value SNR	Liver Spleen Pancreas	–
**C. Park **[22]	Oct 2020		RS	37	ASIR-V 80//100% DLR-L/M/H	GE Healthcare	CT value Noise SNR/CNR	Liver	$\frac{{ROI}_{\mathrm{organ}}-{ROI}_{\mathrm{fat}}}{\mathrm{noise}}$
**R. Singh **[23]	Sept 2019		PS	59	FBP AIDR3D FIRST AiCE	Canon Medical Systems	Noise SNR	Liver	–
**A. Steuwe **[24]	Jan 2021		RS	27	FBP SAFIRE DLR	Siemens Healthineers	CT value Noise SNR/CNR	Liver Spleen	$\frac{{ROI}_{\mathrm{organ}}-{ROI}_{\mathrm{fat}}}{\mathrm{noise}}$
**X. Wang **[25]	April 2021		PS	251	FBP 30% IR DLR 50%/100%	Neusoft Medical	Noise SNR/CNR	Liver	$\frac{{2\left({ROI}_{\mathrm{organ}}-{ROI}_{\mathrm{background}}\right)}^{2}}{{{SD}_{\mathrm{organ}}}^{2}+{{SD}_{\mathrm{background}}}^{2}}$
**L. Zeng **[26]	Feb 2021		PS	207	HIR 50% DLR	United Imaging Healthcare	CT value Noise SNR/CNR	Liver	$\frac{{ROI}_{\mathrm{organ}}-{ROI}_{\mathrm{muscle}}}{\mathrm{noise}}$

*PS* prospective, *RS* retrospective, *AIDR3D* adaptive iterative dose reduction 3D, *FIRST* forward-projected model-based iterative reconstruction solution, *AiCE* advanced intelligent clear-IQ engine, *DLR-L/-M/-H* deep learning reconstruction low/medium/high, *ASIR-V* adaptive statistical iterative reconstruction, *FBP* filtered back-projection, *SAFIRE* sinogram affirmed iterative reconstruction, *IR* iterative reconstruction, *SNR* signal-to-noise ratio, *CNR* contrast-to-noise ratio

Per study the formula of CNR is shown in Table 1. All 6 articles included in the meta-analysis tried to determine image quality of hybrid IR and DLR. Study goals showed similarity and are shown in Table 1.

In all 6 studies, image quality was assessed independently by two radiologists blinded for image reconstruction. Slice thickness varied between 1.25 and 5.0 mm. ROI placement was linked for different reconstruction techniques in three of six articles.

### Quality assessment

The results of the quality assessment are summarized in Table 2. The complete overview of the specific points given to the studies can be found in Appendix B. All included studies reported the CT scan protocol. In addition, all studies compared DLR to a hybrid technique. Four studies compared FBP to DLR. A total of 12 studies were rated as high quality and 4 studies as moderate quality.

**Table 2:** Quality assessment of included studies

Study	Study design	Data collection	Samples	Analysis	Funding	CT protocol	DLR vs FBP/IR	ROI	Total
Akagi 2019	0	1	4	3	1	2	1	1	13
Akagi 2020	0	1	4	2	1	2	1	0	11
Chao 2021	1	1	3	2	1	2	1	2	13
Ichikawa 2021	0	1	4	2	0	2	1	1	11
Jensen 2020	0	1	4	2	0	2	1	2	12
Kaga 2021	1	1	4	2	0	2	1	0	11
Kim 2021	0	1	4	3	0	2	1	1	12
Li 2021	1	1	4	2	0	2	2	1	13
Nakamura 2019	0	1	4	2	0	2	1	1	11
Nakamura 2020	0	1	5	2	1	2	1	0	12
Noda 2021	1	1	4	3	0	2	1	2	14
Park 2020	0	1	4	3	1	2	1	0	12
Singh 2019	1	1	4	2	0	2	2	1	13
Steuwe 2021	0	1	3	3	0	2	2	2	13
Wang 2021	1	1	5	2	1	2	2	2	16
Zeng 2021	1	1	6	2	0	2	1	2	15

*DLR* deep learning reconstruction, *FBP* filtered back-projection, *IR* iterative reconstruction, *ROI* region of interest

### Descriptive results

Kaga et al. and Park et al. showed a statistically significantly higher mean CT value in liver tissue in DLR than in hybrid IR [16, 22]. However, these differences were clinically not relevant with maximum differences of 0.5–0.6 HU. All other studies that were included in the meta-analysis found no significant difference between reconstruction techniques in CT values, measured in the liver, spleen, pancreas, or renal cortex. Kim et al. pointed out that CT values of DLR images were more likely to be higher than those of hybrid IR [17].

Akagi et al.(2019), Li et al., Steuwe et al., and Wang et al.compared FBP to DLR [12, 18, 24, 25]. The difference in mean CT value found in these studies was not significant in abdominal organs.

Steuwe et al.described that DLR was significantly different in the comparison between DLR and IR (*p* = 0.007) in spleen tissue; other abdominal tissues were not significantly different between these reconstruction techniques. The average CT values for liver, spleen, and fat tissue obtained by the three reconstruction techniques (FBP, IR, and DLR) were within ± 0.3 HU (absolute difference) and ± 1.3% (relative difference) [24]. Zeng et al.also showed no statistical differences in mean CT values of liver and fat tissues between low-dose DLIR and low-dose IR (mean CT value difference was 0.3 HU (*p* = 0.837) and 1.8 HU (*p* = 0.118), respectively) [26].

The 14 articles that compared CNRs of DLR with CNRs of FBP or hybrid IR all showed a significantly higher CNR for DLR. While Wang et al.showed no significant difference in SNR between 50% DLR and hybrid IR in obese patients [25], all other SNRs were significantly higher for DLR. The other 8 articles mentioning SNR showed significantly higher SNRs for all examined organs.

### Quantitative results

Six articles showed CT values of GE Healthcare’s ASiR-V 30–50% (hybrid IR) and TrueFidelity (DLR) reconstruction techniques, so these papers were included in the meta-analysis [13, 15–18, 21]. CT values were analyzed for the liver, spleen, and pancreas. All six studies examined the liver, five included the spleen, and only two studies measured CT values in the pancreas.

**Fig. 2:**
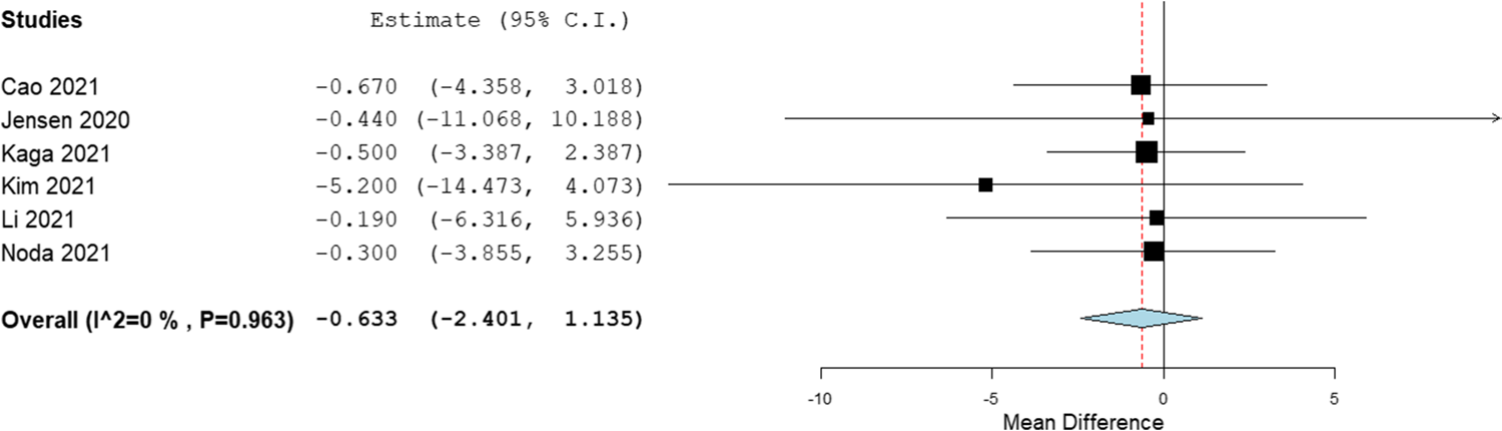
Forest plot of the mean CT value difference (95% CI) in the liver

**Fig. 3:**
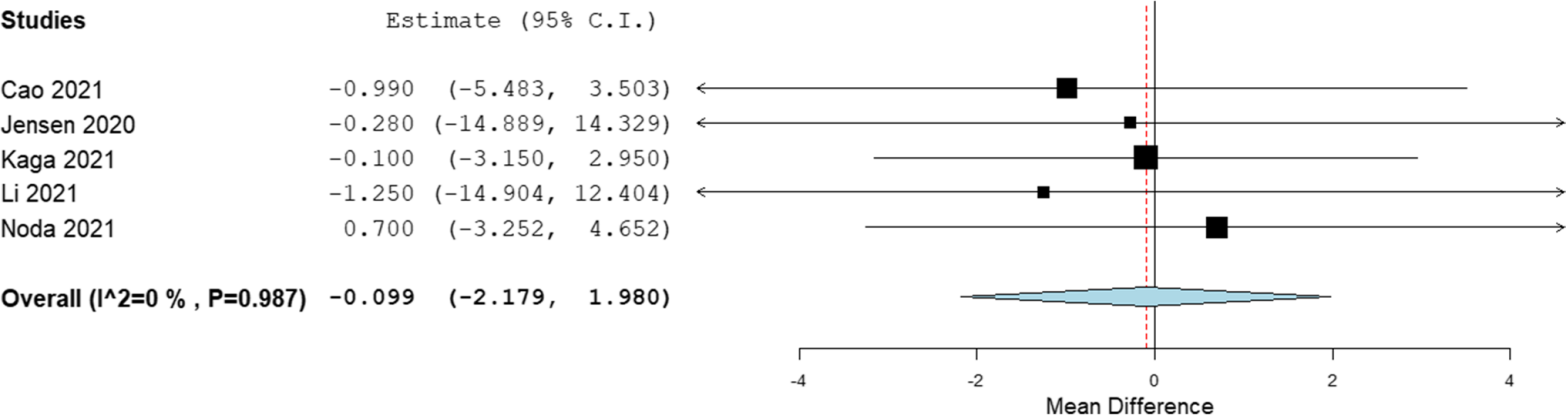
Forest plot of the mean CT value difference (95% CI) in the spleen

**Fig. 4:**
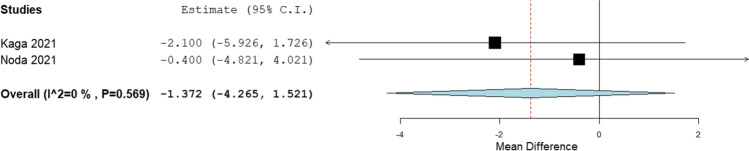
Forest plot of the mean CT value difference (95% CI) in the pancreas

In the liver, the mean difference between hybrid IR and DLR was − 0.633 HU (*p* = 0.483, SD ± 0.902 HU) (Fig. 2). In the spleen, the mean difference between hybrid IR and DLR was − 0.099 HU (*p* = 0.925, SD ± 1.061 HU) (Fig. 3). In the pancreas, the mean difference between hybrid IR and DLR was − 1.372 HU (*p* = 0.353, SD ± 1.476 HU) (Fig. 4). No significant differences were found. All meta-analyses showed low heterogeneity (*I*^2^ = 0%).

Five of five studies that described diagnostic confidence in the liver showed significantly higher confidence of GE Healthcare’s DLR than hybrid IR, rated by experienced radiologists. Results on the five-point scale of the comparison of hybrid IR vs DLR were as follows: (3.75 vs 4.0, *p* < 0.05) Cao et al. [13], (4.20 vs 4.72, *p* < 0.05) Jensen et al.[15], (3.92 vs 4.29, *p* < 0.0001) Kaga et al.[16], (2.98 vs 3.59, *p* < 0.002) Li et al.[18], and (3.1 vs 4.1, *p* < 0.001) Noda et al.[21].

## Discussion

The results, despite being insignificant, showed a trend of the mean CT values reconstructed by DLR being higher than those reconstructed by hybrid IR. Kim et al. even showed a mean difference of − 5.2 HU, regarding the liver [17]. However, this trend can only be applied to GE Healthcare’s reconstruction techniques. More research is needed to determine this for other vendors. DLR resulted in significantly higher CNRs and SNRs compared to hybrid IR and FBP. Results on subjective image quality are in favour of DLR. The higher diagnostic confidence in the liver rated by experienced radiologists is promising. However, more research on diagnostic confidence for other abdominal organs needs to be conducted to provide a complete overview.

Only Noda et al. reported a lower CT value with DLR in the spleen, but differences were also insignificant [21]. During the literature search, one study (Matsukiyo et al. [27]) was excluded because this study assessed the abdominal arteries and no organs. However, hybrid IR and DLR were compared, and this study showed a significant difference in reconstructed CT values of two arteries. Here, the CT values of images reconstructed by DLR were significantly higher. This study used a Canon Medical Systems Corporation CT scanner. Small differences in scan protocol existed between the included studies for our meta-analysis but did not affect CT value comparison as the tube voltages of all studies were similar.

Unfortunately, there were only a few articles included that compared DLR to FBP [18, 23–25]. For that reason, no meta-analysis of these reconstruction techniques could be performed. All articles that included FBP described that image quality of DLR was better than FBP and IR. Most articles studied differences in SNR, CNR, and lesion detection between DLR and other reconstruction techniques and concluded that DLR works very well in low-dose CT imaging. With better image quality and no difference in CT values between other reconstruction techniques, DLR is recommended for clinical diagnosis based on CT densitometry.

An important aspect of DLR is that its performance highly depends on the dataset used for training the algorithm. A heterogeneous dataset representative for all patients is required to prove accuracy for all patient groups. One study was found which was conducted in Europe [24]. Other studies were conducted in East Asia (*n* = 13) [11–14, 16–22, 25, 26] and North America (*n* = 2) [15, 23]. In East-Asian studies, the average weight of the patient group tends to be lower than in other regions in the world. Due to a higher obesity rate in western cultures, beam hardening can influence the image reconstruction more in these cultures and therefore the accuracy of CT values.

Hybrid IR data were used for our meta-analysis if the hybrid level was between 30 and 50%. This range represents the hybrid IR technique best. A higher level will result in a higher percentage model-based IR technique which was not the intended reconstruction technique.

Differences between the studies included in the meta-analysis existed in methodology of image assessment. Slice thickness could affect SNR, CNR, and diagnostic confidence as increasing slice thickness results in lower noise levels. ROI shape and size were not similar and could also affect SNR, CNR, and diagnostic confidence.

In all included studies [11–16, 18–23, 26] , except for Kim et al., Steuwe et al., and Wang et al. [17, 24, 25], contrast-enhanced CT was used. In contrast-enhanced CT, a difference in CT value per scan or reconstruction technique is less relevant compared to non-contrast CT, because of the small CT value range in non-contrast CT. When this range is small, it is more important to conduct accurate CT values.

PixelShine described by Steuwe et al. [24] is not developed by CT vendors, while all other reconstruction techniques are. Even though PixelShine reduces image noise by using a deep learning algorithm, it is not clear if the image is reconstructed from the raw data or that the algorithm uses FBP or IR reconstructed images as input. On top of that, this reconstruction technique is not yet approved by the Food and Drug Administration (FDA) [6].

No phantom studies were included in this systematic review because the phantoms lacked abdominal characteristics. Phantom studies can, however, be useful in determining the uniformity and validity of CT values of a single reconstruction technique. CT uniformity dictates that for scanning a uniform material, the mean CT value does not depend on the position within the image. This is important when evaluating the accuracy of a reconstruction technique. More research on uniformity and validity is needed for an even better comparison between reconstruction techniques. Phantom studies can also be used to determine whether variable tube currents or tube voltages affect CT values.

In conclusion, this systematic review and meta-analysis identified no significant difference in CT values reconstructed by FBP, hybrid IR, and DLR in abdominal organs. This shows that these reconstruction techniques are consistent in reconstructing CT values and can thus be applied safely in the clinical setting. Also, DLR images showed a significantly higher CNR and SNR than FBP and hybrid IR images. However, there was a limited number of studies that described FBP, and the meta-analysis was only done with studies that described GE’s hybrid IR and DLR. Therefore, more research is needed to determine whether the same conclusion is true for the comparison between FBP and DLR and other CT vendors.

## Supplementary Information

Below is the link to the electronic supplementary material.

Supplementary file1 (DOCX 26 KB)

## Abbreviations

CNN: Convolutional neural networks
CNR: Contrast-to-noise ratio
DLR: Deep learning reconstruction
FBP: Filtered back-projection
HU: Hounsfield unit
IR: Iterative reconstruction
MBIR: Model-based iterative reconstruction
ROI: Region of interest
SNR: Signal-to-noise ratio

## Declarations

### Guarantor

The scientific guarantor of this publication is Aart J. van der Molen, Leiden UMC, the Netherlands.

### Statistics and Biometry

No complex statistical methods were necessary for this paper.

### Study subjects or cohorts overlap

All the included studies have been previously reported as an original research paper.

### Methodology

- Systematic Review
- Meta-analysis
