# Supplementary material for: The effect of deep learning reconstruction on abdominal CT densitometry and image quality: a systematic review and meta-analysis
Source: Eur Radiol. 2021 Dec 15;32(5):2921–9. doi: 10.1007/s00330-021-08438-z (PMC9038933; doi:10.1007/s00330-021-08438-z)
Supplement: Supplementary file 1 — Supplementary file1 (DOCX 26 KB) [file 330_2021_8438_MOESM1_ESM.docx]

**Appendix A**

**Search string**

**PubMed; conducted 10-5-2021: 178 results**

(("CT densitometry"[tw] OR "CT densitometric"[tw] OR "CT densitometr*"[tw] OR "computer tomographic densitometry"[tw] OR "computed tomographic densitometry"[tw] OR "computed tomography densitometry"[tw] OR "qCT" [tw] OR (("Densitometry"[mesh] OR "densitometry"[tw] OR "densitometric"[tw] OR "densitometr*"[tw] OR ("densit*"[tw] AND "measurement*"[tw])) AND ("Tomography, X-Ray Computed"[mesh] OR "CT"[tw] OR "computer tomographic"[tw] OR "computed tomographic"[tw] OR "computed tomography"[tw] OR "computer tomography"[tw] OR "computer assisted tomographic"[tw] OR "computed assisted tomography"[tw] OR "computer assisted tomography"[tw]))) AND ("Hounsfield Unit"[tw] OR "Hounsfield Units"[tw] OR "Houndsfield Unit"[tw] OR "Houndsfield Units"[tw] OR (("Hounsfield"[tw] OR "Houndsfield"[tw]) AND ("Unit"[tw] OR "Units"[tw] OR "HU"[tw] OR "HUs"[tw])) OR "Hounsfield "[tw] OR "Hounsfield*"[tw] OR "Houndsfield"[tw] OR "Houndsfield*"[tw] OR "HU"[tw] OR "HUs"[tw] OR "CT value"[tw] OR "CT values"[tw] OR "CT-value"[tw] OR "CT-values"[tw] OR "CT number"[tw] OR "CT numbers"[tw] OR "CT-number"[tw] OR "CT-numbers"[tw]) AND ("filtered back projection"[tw] OR "filtered back projections"[tw] OR "filtered backprojection"[tw] OR "filtered backprojections"[tw] OR "filter back projection"[tw] OR ("filter*"[tw] AND ("backprojec*"[tw] OR "back project*"[tw]) AND "reconstruc*"[tw]) OR "FBP"[tw] OR "iterative reconstruction"[tw] OR "iterative reconstruct*"[tw] OR ("iterat*"[tw] AND "reconstruct*"[tw]) OR "IR"[tw] OR "MBIR"[tw] OR ("model-based"[tw] AND ("iterative reconstruction"[tw] OR "IR"[tw])) OR (("AIDR3D"[tw] OR "Forward Projected Model-based Iterative Reconstruction SoluTion"[tw] OR "Advanced Modeled Iterative Reconstruction"[tw] OR "iDose"[tw] OR "ASIR"[tw]) AND "reconstruct*"[tw])) OR ((("AI"[tw] OR "artificial intelligence"[tw] OR "deep learning"[tw] OR "DLR"[tw]) AND ("reconstruct*"[tw] OR "iterat*"[tw]))) OR "DLIR"[tw] OR (("AICE"[tw] OR "TrueFidelity"[tw]) AND "reconstruct*"[tw]) OR ("Convolutional neural networks"[tw] OR "CNN"[tw] AND ("reconstruct*"[tw] OR "iterat*"[tw])) AND ("Abdomen"[tw] OR "abdominal"[tw] OR "abdominal imaging"[tw] OR "adrenal glands"[tw] OR "kidneys"[tw] OR "renal"[tw] OR "liver"[tw] OR "hepatic"[tw] OR "Abdominal Cavity"[Mesh] OR "Abdomen"[Mesh] OR "Kidney"[mesh] OR "kidney"[tw] OR "Liver"[mesh] OR "Kidney Diseases"[mesh] OR "Liver Diseases"[mesh] OR "hepatic"[tw] OR "Adrenal Glands"[Mesh] OR "Adrenal Glands"[tw] OR "Adrenal Gland Diseases"[Mesh] OR "Adrenal*"[tw]) NOT ("Magnetic Resonance Imaging"[majr] OR "magnetic resonance"[ti] OR "MR imag*"[ti] OR "MRI"[ti]))

**Embase; conducted 10-5-2021: 148 results**

((CT densitometry.mp OR CT densitometric.mp OR CT densitometr*.mp OR computer tomographic densitometry.mp OR computed tomographic densitometry.mp OR computed tomography densitometry.mp OR qCT.mp OR ((exp Densitometry/ OR densitometry.mp OR densitometric.mp OR densitometr*.mp OR (densit*.mp AND measurement*.mp)) AND (exp Tomography, X-Ray Computed/ OR CT.mp OR computer tomographic.mp OR computed tomographic.mp OR computed tomography.mp OR computer tomography.mp OR computer assisted tomographic.mp OR computed assisted tomography.mp OR computer assisted tomography.mp))) AND (Hounsfield Unit.mp OR Hounsfield Units.mp OR Houndsfield Unit.mp OR Houndsfield Units.mp OR ((Hounsfield.mp OR Houndsfield.mp) AND (Unit.mp OR Units.mp OR HU.mp OR HUs.mp)) OR Hounsfield .mp OR Hounsfield*.mp OR Houndsfield.mp OR Houndsfield*.mp OR HU.mp OR HUs.mp OR CT value.mp OR CT values.mp OR CT-value.mp OR CT-values.mp OR CT number.mp OR CT numbers.mp OR CT-number.mp OR CT-numbers.mp) AND (filtered back projection.mp OR filtered back projections.mp OR filtered backprojection.mp OR filtered backprojections.mp OR filter back projection.mp OR (filter*.mp AND (backprojec*.mp OR back project*.mp) AND reconstruc*.mp) OR FBP.mp OR iterative reconstruction.mp OR iterative reconstruct*.mp OR (iterat*.mp AND reconstruct*.mp) OR IR.mp OR MBIR.mp OR (model-based.mp AND (iterative reconstruction.mp OR IR.mp)) OR ((AIDR3D.mp OR Forward Projected Model-based Iterative Reconstruction SoluTion.mp OR Advanced Modeled Iterative Reconstruction.mp OR iDose.mp OR ASIR.mp) AND reconstruct*.mp)) OR (((AI.mp OR artificial intelligence.mp OR deep learning.mp OR DLR.mp) AND (reconstruct*.mp OR iterat*.mp))) OR DLIR.mp OR ((AICE.mp OR TrueFidelity.mp) AND reconstruct*.mp) OR (Convolutional neural networks.mp OR CNN.mp AND (reconstruct*.mp OR iterat*.mp)) AND (Abdomen.mp OR abdominal.mp OR abdominal imaging.mp OR adrenal glands.mp OR kidneys.mp OR renal.mp OR liver.mp OR hepatic.mp OR exp Abdominal Cavity/ OR exp Abdomen/ OR exp Kidney/ OR kidney.mp OR exp Liver/ OR exp Kidney Diseases/ OR exp Liver Diseases/ OR hepatic.mp OR exp Adrenal Glands/ OR Adrenal Glands.mp OR exp Adrenal Gland Diseases/ OR Adrenal*.mp) NOT (exp Magnetic Resonance Imaging/ OR magnetic resonance.ti OR MR imag*.ti OR MRI.ti)) NOT (conference review or conference abstract).pt

**Appendix B**
**Quality Assessment**

1. **The study was conducted prospectively**
2. **The data was collected first-hand**
3. **a) Number of patients was described**
   **b) Anatomical location where data was collected was described**
   **c) Samples > 10**
   **d) Samples > 40**
   **e) Samples > 60**
   **f) Samples > 100**
4. **a) Statistical analysis was performed**
   **b) Multiple test correction was done**
   **c) Results contained descriptive statistic results**
5. **Funding was described**
6. **a) CT scanner type was described**
   **b) CT scan protocol was described**
7. **a) DLR was compared with IR**
   **b) DLR was compared with FBP**
8. **a) Images were linked for ROI placement**
   **b) Size of ROI was described**

|  | Akagi 2019 | Akagi 2020 | Cao 2021 | Ichikiwa 2021 | Jensen 2020 | Kaga 2021 | Kim 2021 | Li 2021 | Nakamura 2019 | Nakamura 2020 | Noda 2021 | Park 2020 | Singh 2019 | Steuwe 2021 | Wang 2021 | Zeng 2021 |
| --- | --- | --- | --- | --- | --- | --- | --- | --- | --- | --- | --- | --- | --- | --- | --- | --- |
| 1. | 0 | 0 | 1 | 0 | 0 | 1 | 0 | 1 | 0 | 0 | 1 | 0 | 1 | 0 | 1 | 1 |
| 2. | 1 | 1 | 1 | 1 | 1 | 1 | 1 | 1 | 1 | 1 | 1 | 1 | 1 | 1 | 1 | 1 |
| 3a. | 1 | 1 | 1 | 1 | 1 | 1 | 1 | 1 | 1 | 1 | 1 | 1 | 1 | 1 | 1 | 1 |
| 3b. | 1 | 1 | 1 | 1 | 1 | 1 | 1 | 1 | 1 | 1 | 1 | 1 | 1 | 1 | 1 | 1 |
| 3c. | 1 | 1 | 1 | 1 | 1 | 1 | 1 | 1 | 1 | 1 | 1 | 1 | 1 | 1 | 1 | 1 |
| 3d. | 1 | 1 | 0 | 1 | 1 | 1 | 1 | 1 | 1 | 1 | 1 | 1 | 1 | 0 | 1 | 1 |
| 3e. | 0 | 0 | 0 | 0 | 0 | 0 | 0 | 0 | 0 | 1 | 0 | 0 | 0 | 0 | 1 | 1 |
| 3f. | 0 | 0 | 0 | 0 | 0 | 0 | 0 | 0 | 0 | 0 | 0 | 0 | 0 | 0 | 1 | 1 |
| 4a. | 1 | 1 | 1 | 1 | 1 | 1 | 1 | 1 | 1 | 1 | 1 | 1 | 1 | 1 | 1 | 1 |
| 4b. | 1 | 0 | 0 | 0 | 0 | 0 | 1 | 0 | 0 | 0 | 1 | 1 | 0 | 1 | 0 | 0 |
| 4c. | 1 | 1 | 1 | 1 | 1 | 1 | 1 | 1 | 1 | 1 | 1 | 1 | 1 | 1 | 1 | 1 |
| 5. | 1 | 1 | 1 | 0 | 0 | 0 | 0 | 0 | 0 | 1 | 0 | 1 | 0 | 0 | 1 | 0 |
| 6a. | 1 | 1 | 1 | 1 | 1 | 1 | 1 | 1 | 1 | 1 | 1 | 1 | 1 | 1 | 1 | 1 |
| 6b. | 1 | 1 | 1 | 1 | 1 | 1 | 1 | 1 | 1 | 1 | 1 | 1 | 1 | 1 | 1 | 1 |
| 7a. | 1 | 1 | 1 | 1 | 1 | 1 | 1 | 1 | 1 | 1 | 1 | 1 | 1 | 1 | 1 | 1 |
| 7b. | 0 | 0 | 0 | 0 | 0 | 0 | 0 | 1 | 0 | 0 | 0 | 0 | 1 | 1 | 1 | 0 |
| 8a. | 0 | 0 | 1 | 0 | 1 | 0 | 0 | 0 | 0 | 0 | 1 | 0 | 0 | 1 | 1 | 1 |
| 8b. | 1 | 0 | 1 | 1 | 1 | 0 | 1 | 1 | 1 | 0 | 1 | 0 | 1 | 1 | 1 | 1 |
| Total | 13 | 11 | 13 | 11 | 12 | 11 | 12 | 13 | 11 | 12 | 14 | 12 | 13 | 13 | 17 | 15 |
